# Identifying risk factors for drug use recurrence with ecological momentary assessment, wearable technologies, and machine learning: a feasibility trial of peer recovery support specialist intervention

**DOI:** 10.3389/fdgth.2026.1744937

**Published:** 2026-07-03

**Authors:** James J. Mahoney III, Victor S. Finomore, Jennifer L. Marton, Lucinda J. England, Sara McFoy, Danielle Romanoff, Jad Ramadan, Anahita Zarei, Amer Mahyoub, Jessie Crooks, James H. Berry, Steven D. Shirk, Manish Ranjan, Ali R. Rezai

**Affiliations:** 1 Department of Psychiatry and Neurobehavioral Sciences, University of Virginia School of Medicine, Charlottesville, VA, United States; 2 Department of Behavioral Medicine and Psychiatry, Rockefeller Neuroscience Institute, West Virginia University School of Medicine, Morgantown, WV, United States; 3 Department of Neuroscience, Rockefeller Neuroscience Institute, West Virginia University School of Medicine, Morgantown, WV, United States; 4 Department of Neurosurgery, Rockefeller Neuroscience Institute, West Virginia University School of Medicine, Morgantown, WV, United States

**Keywords:** drug use recurrence, ecological momentary assessment, overdose, peer recovery support specialist, relapse, substance use disorder, wearable technology

## Abstract

**Background:**

Identifying predictors of relapse/drug use recurrence (DUR) in real-time could allow the rapid implementation of overdose prevention interventions for those with substance use disorders (SUD). Wearable devices and phone-based applications for self-reported assessments in the patient's natural environment [e.g., ecological momentary assessment (EMA)] have potential for predicting DUR. Peer recovery support specialists (PRSS) play a critical role in reducing DUR risk. The benefits of utilizing PRSS resources in response to alerts derived from wearable technology and EMA data are unknown. This feasibility study investigates a) the use of wearable technologies/EMA to predict physiological/behavioral biomarkers of DUR and b) opportunities for PRSS-based interventions based on predictions.

**Methods:**

Participants were recruited from various settings (e.g., SUD treatment, sober living), were provided a commercial wearable device (Oura ring), and were prompted daily to complete an EMA application assessing mood and substance cravings. Participants were monitored for 90 days (Baseline Phase), then randomized to the Standard of Care (SoC) or PRSS Intervention arm and followed for two 90-day phases. When machine learning algorithms detected an anomaly, an alert was sent to the participant's phone. The PRSS was sent an alert to contact participants in the PRSS Intervention arm.

**Results:**

Of 229 participants enrolled, 108 provided EMA and Oura data for ≥1 of 90 days during Baseline, Phase 1, and Phase 2; 63 provided ≥30% of the data across the 3 phases. Twenty-six were randomized to the PRSS Intervention arm, and 37 were randomized to the SoC arm. The PRSS made 483 call attempts for unique alerts; the average per participant across the study was ∼20 (median = 15; range = 1–53). The PRSS intervention arm had modest but significant decreases in anxiety, stress, depression, angst composite (all *p*'s < 0.001), and maximum craving (*p* = 0.011) relative to the SoC arm.

**Discussion:**

This feasibility study highlights the potential benefits and barriers for using models to predict risk factors for DUR via wearable devices and EMA and supports the utility of a PRSS intervention once the model detects an elevated risk for DUR. Patient compliance and attrition must be improved to optimize this approach as a clinical tool. We discuss challenges and recommend strategies for future studies.

## 1 Introduction

According to the National Survey on Drug Use and Health, it is estimated that over 48.4 million individuals in the United States (U.S.) met criteria for a substance use disorder (SUD) (28.2 million with a drug use disorder, 27.9 million with an alcohol use disorder, and 7.7 million with both) (1). While there was an overall decrease in overdose deaths in the U.S. in 2024, the number remains elevated at 87,000 overdose-related fatalities annually, the majority of which involve opioids (2). Overall, it is estimated that in 2024 there were 40.7 million individuals with SUD who did not receive any form of SUD treatment (1). Even though medications for opioid use disorder (MOUD) reduce overdose deaths and overall mortality (3), only 17% of individuals with OUD received treatment with MOUD in 2024 (1). Thus, there is an urgent need to engage more individuals in SUD treatment programs, especially given the high rates of attrition with current Standard of Care (SoC) treatments (4, 5).

Individuals with OUD are at an especially elevated risk of overdose following periods of abstinence due to lowered drug tolerance (such as following residential treatment or incarceration) (6). Other transitional periods that can increase overdose risk include disruptions in treatment or in social support, during times of financial and housing instability, and during acute exacerbations of stress (7, 8). Providing supplemental support services to individuals during high-risk periods could reduce the number of overdose deaths (9). There is a growing interest in the integration of peer recovery services (nonclinical assistance supporting recovery from SUD through emotional, informational, instrumental, and affiliational services) into routine SUD treatment using peer recovery support specialists (PRSS) (10). Traditionally, PRSSs are individuals with lived experience of addiction who have achieved sustained abstinence and have received specific training in assisting others in recovery. Services include serving as mentors and role models, educating those with SUD and those in the community about substance use and substance use recovery, and linking individuals to resources like housing, work, education, transportation, and childcare (10). PRSSs can also facilitate collaboration within care teams and the benefits of providing support through PRSSs include increased engagement in SUD treatment, increased participation in 12-step programs, and reduced substance use (10–12).

The utilization of PRSSs could help decrease the risk of drug use recurrence (DUR) and overdose in individuals, especially during high-risk transition periods. There are several tools that could potentially be made available to PRSSs to help with this endeavor. For example, wearable devices that continuously monitor biometric signals [e.g., heart rate/variability (HR/HRV), sleep characteristics] have been used to detect physiological markers associated with DUR (13–18). Similarly, phone-based applications for obtaining self-reported assessments in the patient's natural environment [ecological momentary assessment (EMA)] have been used to identify self-reported predictors of DUR, such as experiencing triggers or increased isolation (16, 18, 19). These technologies could help PRSSs recognize near-term DUR and thus help reduce risk of DUR and overdose. A previous pilot study conducted by our team investigated the utilization of wearable biosensors combined with EMA in individuals with OUD and co-occurring other SUDs who were enrolled in treatment programs. 77 participants wore a smartwatch (Garmin Vívosmart), which continuously monitored several physiological variables (heart rate, HRV, blood oxygen saturation, stress, activity, and sleep) and also submitted daily EMA feedback on cravings, mood, and behavior using a smartphone application (15). Of the 77 participants enrolled, 34 (44%) participants experienced a DUR event during the study period. For individuals with a DUR, the smartwatch “stress score” (derived from HRV) indicated that HRV was significantly lower during the week prior to and the week of the DUR relative to the weeks of abstinence. In a separate study of 30 individuals with SUD, the authors found that future stress and craving could be predicted accurately ∼70%–75% of the time (18). Taken together, these results suggest that data acquired via wearable technologies and *EMA could help PRSSs and clinical teams predict near-term DUR, prompting an intervention before drug use actually occurs.*

In the current study, we expand upon our previous pilot study by examining the feasibility of conducting long-term monitoring of biometric and self-reported data in participants with OUD in various stages of recovery. The first goal was to develop personalized and generalized predictive models to detect aberrant patterns—defined as deviations from expected self-reported or biometric responses (e.g., increased craving, anxiety). The second goal was to assess the utility and feasibility of predictive models to use in a real-world setting by alerting a PRSS research team member, who then attempted to contact those participants with predicted increases in craving/anxiety ratings. Preliminary data were obtained related to the PRSS intervention on self-reported anxiety, depression, stress, and craving; trends over time were compared between participants who were randomized to receive calls (the PRSS Intervention arm) vs. those who did not receive calls [the SoC arm]. Lessons learned from this study will be applied to future efforts to incorporate biometric monitoring and EMA into routine care for those in recovery.

## 2 Methods

### 2.1 Study design

**Figure 1:**
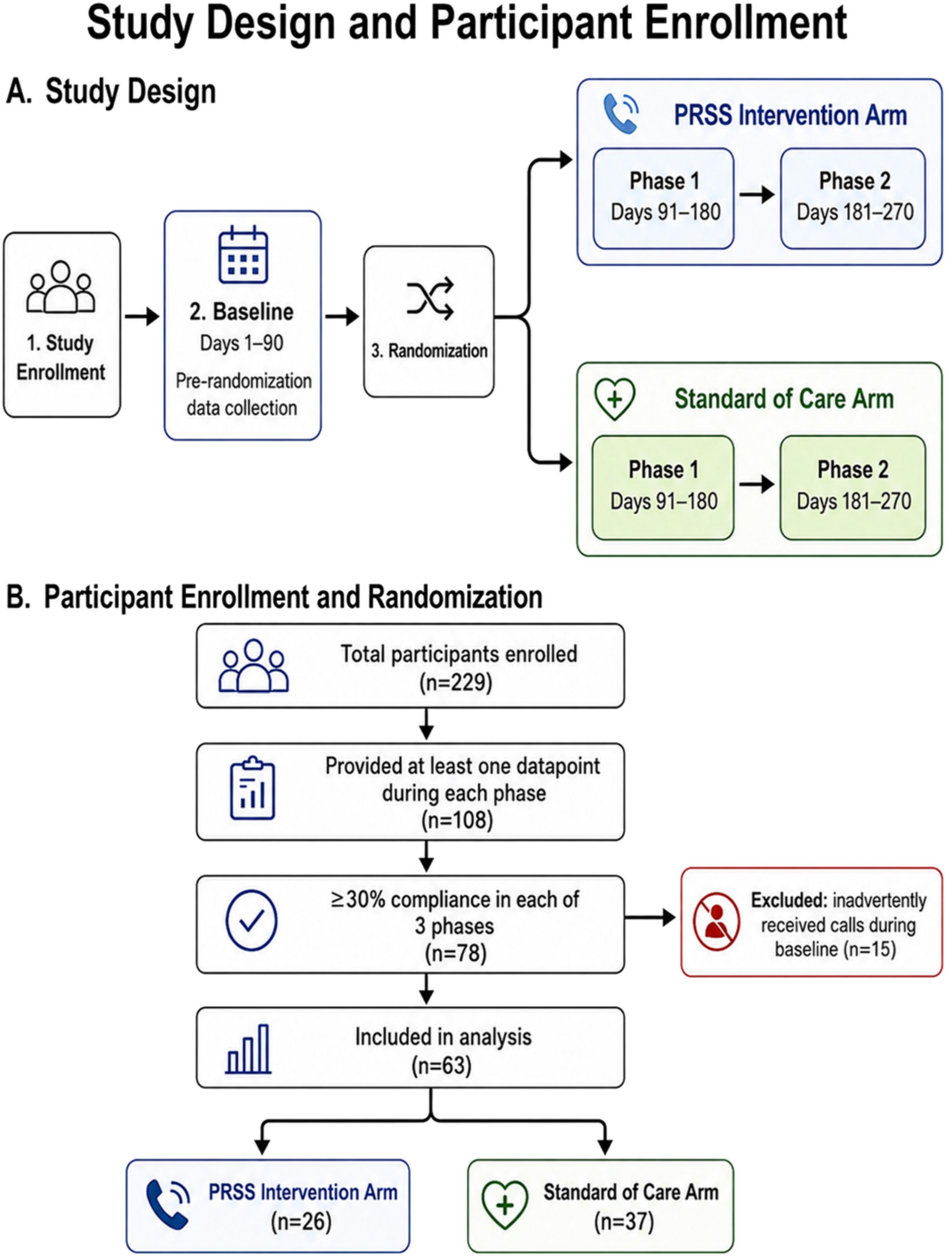
**(A)** Study design; **(B)** participant enrollment and randomization.

After signing consent and being deemed eligible, participants were fitted with the wearable device [Oura ring (Generation 3)] which they were instructed to wear continuously. The Oura app and Rockefeller Neuroscience Institute (RNI) Health app (described below) were then downloaded to participants' phones. All participants were monitored for three months (the Baseline Phase) and then randomized to the SoC arm or the PRSS Intervention arm. Randomized participants were followed for four three-month phases. Attrition rates following the second phase were high; therefore, this report will only discuss findings of the Baseline Phase (Days 1–90), Phase 1 (Days 91–180), and Phase 2 (Days 181–270). The study design flow diagram is shown in Figure 1A.

### 2.2 Participants

Participants represent a convenience sample and were recruited from a variety of local settings, including residential and outpatient SUD treatment programs, and sober living environments. Prior to consent, a PRSS from the study research team met with interested participants and provided them with information about the study and answered questions. Inclusion criteria required participants to be 18 years of age or older, have a current or past diagnosis of SUD, be able to download the RNI Health app and wearable device app onto their smart device, speak English, and provide informed consent. All procedures were approved by the Institutional Review Board of the West Virginia University (WVU) School of Medicine. Participant enrollment began in September 2023 and completed in November 2024.

### 2.3 Assessments, measures, and sources of data

#### 2.3.1 Demographic and clinical characteristics

Participant demographic characteristics were obtained from the RNI Health App (described below). For those participants enrolled in the WVU Health system, information was also extracted from electronic medical records and included data such as age at study enrollment, sex, SUD clinic dismissal date/reason (if applicable), and any reported DURs during study enrollment (documented in clinical notes from the participant's psychiatrist or therapist and/or urine toxicology results).

#### 2.3.2 Physiological monitoring

Participants were provided with an Oura Ring (Generation 3; https://www.ouraring.com) for the duration of the study (lost rings were replaced upon request). The Oura Ring is a commercially available wearable device which continuously monitors several different physiological metrics; the following were deemed variables of interest in the current study and were used in the predictive models: HR, HRV, respiratory rate, sleep quality measurements such as sleep stage tracking, duration, efficiency (time asleep divided by time in bed), number of times detected as a “wake up” (wake up count), movement during sleep (restlessness), and the maximum body temperature during sleep (when available). The device connects via Bluetooth to the RNI Health app and the Oura app, where data can be accessed by the user (recorded via smartphone and linked to a de-identified number)*.* Both apps are free to download and is available on both iOS and Android platforms. Participants were instructed to synchronize their Oura ring frequently (daily if possible) so that the data could be transmitted to the Oura cloud.

#### 2.3.3 Ecological momentary assessment (EMA)

The EMA was completed using the RNI Health app, which is a smartphone application developed by the RNI to assist in exchanging information between participants who are enrolled in RNI research studies, research team members, and health care providers. For the current study, this app was used to administer daily assessments and surveys and to deliver individualized content to participants (push alerts) throughout the duration of the study. This app links to de-identified accounts in the RNI Cloud where data is stored.

#### 2.3.4 Participant compliance

Participant compliance was measured via a point system and based on the number of daily questionnaires/assessments completed and the number of days for which data were downloaded from the wearable device. Participants were compensated up to $100 monthly via gift card for their participation, based on overall monthly compliance.

### 2.4 Development of personalized and generalized models for anomaly detection

Four personalized computer models to predict an individual's risk for craving, anxiety, stress, and depression were developed using deep learning (machine learning) architectures. Unlike generic approaches, each individual's model is trained specifically from their own data, allowing the understanding of their unique patterns and responses. The system then monitors these predictions in real time. If an individual's predicted risk score increases substantially (defined as >1.5 standard deviations from their previous 28-day average), a personalized push alert is triggered. The intention is that by developing an early “warning system” via the personalized push alert, individuals and their healthcare providers can proactively address potential issues before they become more severe. In addition to the personalized models, four generalized models were also developed to predict risk for craving, anxiety, stress, and depression. Unlike the personalized models, which are trained exclusively on an individual's own data to capture their unique patterns, the generalized models are trained on combined data from the broader study sample. These generalized models provide risk predictions that reflect group-level trends rather than individual-specific norms. They serve as the default option for participants who do not yet have enough personal data to support the development of a reliable personalized model. The detailed process for how this system was developed, implemented, and deployed can be found in the Supplementary Materials.

### 2.5 Push-alert notifications and PRSS intervention

#### 2.5.1 Push-alert notifications

For both study arms (SoC and PRSS Intervention), an alert (referred to as a “push alert”) was generated when an anomaly was detected by the machine learning algorithms described above, and a notification was sent to the participant's phone suggesting they contact someone in their support system (e.g., family member, friend, sponsor, etc.). For those randomized to the PRSS Intervention arm, the PRSS was also sent an alert with the participant's identification number, prompting the PRSS to contact the participant by telephone.

#### 2.5.2 PRSS intervention

Once the PRSS received the alert with the participant's identification number, the PRSS attempted to contact the participant via phone to determine their status and to inquire whether the participant needed any resources (such as times/locations of AA/NA meetings, sleep and/or relaxation techniques, etc.). The algorithm also randomly generated push alerts for participants in the PRSS Intervention arm when no anomaly was detected (to test whether anomaly-based alerts/calls affected participant outcomes differently than random calls). The “random” alerts were generated at a 1:3 ratio (one random alert for every three anomaly-generated alerts). The PRSS followed the same check-in process with the participants for random alerts as the PRSS was “blinded” to whether the push alert was generated due to an anomaly or was randomly generated.

### 2.6 Statistical analyses

Data related to daily activities (surveys), Oura ring metrics, and various monthly survey scores were compiled and integrated within a Redshift database provided by Amazon Web Services (AWS). This method reduced the need for any additional formatting once data was downloaded. The different data sources were simply merged by matching the appropriate subject ID and date (if necessary) per source. Results were tabulated and the corresponding t-tests and Fisher's Exact Tests were conducted using Python 3.14.0. A portion of the dataset, referred to as the “hold-out” test set, was reserved for evaluating the model's performance on unseen data, helping estimate how well the model will generalize and was not used during model training. The data in the hold-out set was used to validate the model as a comparison to the training set and therefore not included in the training set to maintain an independent comparison without duplication. Hold-out set performance for both the generalized and personalized models was assessed using a “correctness score”, which is a modified version of the mean absolute error (MAE). Since all responses for self-reported anxiety, depression, stress, and craving were on a 0–10 scale, the correctness score leverages this to obtain a “percent correct” metric (rather than a percent error). The denominator of 10 reflects the range of self-report scores. For the generalized model, this score was computed for the entire dataset (i.e., one score for the entire cohort). For the personalized model, this score was computed at the individual level (i.e., one score for each individual).

As previously mentioned in the methods section, when the PRSS received notification of a trigger for an individual, a “check-in” phone call was made to the participant to further investigate. Following the call, the PRSS completed a survey via the RNI Health app recording details of the call, including whether the call was answered, if the participant was safe, what recommendations they gave, whether the participant experienced a DUR or had thoughts of using drugs, and any further notes the PRSS deemed relevant. This survey was also stored in Redshift and was accessible in the same fashion as the EMA.

One of the goals of this study was to evaluate the potential efficacy of the PRSS intervention on reducing self-reported anxiety, depression, stress, and craving levels by comparing their trends over time within participants who received calls to those who did not (the SoC arm). This was accomplished by constructing linear mixed-effects models (LMM) using both study phase, arm, and their interaction as fixed effects and participants as random effects (to account for the lack of independence due to participants submitting repeated measurements). A separate LMM was considered using each of the self-reported anxiety, depression, stress, and maximum craving level (for any of the presented substances) as the response variable. An additional variable, named “angst composite”, was calculated by taking the average of the reported anxiety, depression, and stress scores. Since anxiety, depression, and stress were all discrete numeric variables on a scale of 0–10, the angst composite average resulted in a continuous numeric variable, thus more amenable to the normality assumptions of the LMMs.

Many of the study participants reported very low daily levels of the five variables (anxiety, depression, stress, angst composite, and craving). This created a “floor” effect that biased the analyses since improvement was not possible for participants with responses at or near the minimum value (“0”) at baseline. To overcome this issue, the cohort was split into tertiles for each response variable (the lower third, middle third, and upper third), based on the respective quantiles from the distribution of each variable. These quantiles were created using baseline data, and participants were assigned to a group based on their average at baseline. In other words, if a participant's average was lower than the 33.3% quantile for a specific variable, they were placed into the lower third for that variable. If the average was between the 33.3% and 66.7% quantile, the participant was placed in the middle third, and those with averages larger than the 66.7% quantile were placed in *the upper third for each variable*. The LMMs were then constructed for each group for each variable separately to primarily examine response differences in the higher and middle quantiles (i.e., those less susceptible to floor effects).

## 3 Results

### 3.1 Sample characteristics and compliance

Two hundred twenty-nine participants were enrolled and participated in baseline data collection (daily EMA and physiological monitoring) for 90 days. Demographic information for enrolled participants is described in Table 1. Participants were equally distributed based on sex, were predominantly White (94%), and were 39.7 ± 8.7 (mean ± SD) years of age. Given that a large number of participants were recruited from sober living environments (38%) and residential or outpatient SUD treatment programs (18%), most participants denied recent use of substances in the past 30 days. However, all participants endorsed a past history of using multiple substances, which included cannabis (∼99% of participants reported past use), alcohol (∼94%), cocaine (∼94%), opioids (∼92%), methamphetamine (∼84%), and benzodiazepines (∼67%) (Table 2). Oura and EMA compliance during Baseline, Phase 1, and Phase 2 are detailed in Table 3. For the entire sample of 229 participants, compliance rates declined during each of the three 90-day phases (from ∼41 days during Baseline to ∼30 days during Phase 1 to ∼21 days during Phase 2).

**Table 1:** Participant characteristics.

	All participants enrolled (*n* = 229)^a^	Randomized to PRSS intervention vs. SoC (≥30% compliant)			
		Overall sample (*n* = 63)	PRSS intervention (*n* = 26)	SoC (*n* = 37)	*p*
Age^a^^,^^b^					
Mean (SD)	39.7 (8.7)	40.6 (8.2)	43.6 (10.2)	38.4 (5.7)	0.08
Sex^a^					
Male	105 (49.5%)	30 (52.4%)	15 (57.7%)	15 (40.5%)	0.28
Female	107 (50.5%)	33 (47.6%)	11 (42.3%)	22 (59.5%)	
Race^a^					
White	199 (93.9%)	57 (90.5%)	23 (88.5%)	34 (91.9%)	0.76
Black	4 (1.9%)	1 (1.6%)	0	1 (2.7%)	
Other^c^	9 (4.2%)	5 (7.9%)	3 (11.5%)	2 (5.4%)	
Education^a^					
<12th grade	17 (8.0%)	3 (4.8%)	2 (7.7%)	1 (2.7%)	0.23
High School/GED	77 (36.3%)	27 (42.9%)	13 (50%)	14 (37.8%)	
Some college	72 (34.0%)	22 (34.9%)	7 (26.9%)	15 (40.5%)	
Vocational/technical	23 (10.8%)	6 (9.5%)	4 (15.4%)	2 (5.4%)	
Bachelor's degree	13 (6.1%)	3 (4.8%)	0	3 (8.1%)	
Graduate degree	6 (2.8%)	0	0	0	
Other/No Response	4 (1.9%)	2 (3.2%)	0	2 (5.4%)	
Recruitment Location					
Residential SUD Tx	25 (10.9%)	7 (11.1%)	4 (15.4%)	3 (8.1%)	0.55
Outpatient SUD Tx	15 (6.5%)	4 (6.4%)	3 (11.5%)	1 (2.7%)	
Sober Living	86 (37.6%)	21 (33.3%)	9 (34.6%)	12 (32.4%)	
Comm. Outreach^d^	68 (29.7%)	17 (27.0%)	5 (19.2%)	12 (32.4%)	
Previous Study Pt^e^	35 (15.3%)	14 (22.2%)	5 (19.2%)	9 (24.3%)	

Values represent mean [Standard deviation (SD)] or *n* (%).

Comm., community; PRSS, peer recovery support specialist; Pt, participant; SoC, standard of care; Tx, treatment.

a Of the 229 participants enrolled, 212 completed the demographic survey.

b Age data not obtained from the demographic questionnaire on the app and was extracted for only those with available EMR data [All participants enrolled: *n* = 116; Randomized Overall Sample: *n* = 38 (PRSS Intervention: *n* = 16; SoC: *n* = 22)].

c Indigenous (*n* = 5); Asian (*n* = 1); Not provided (*n* = 3).

d Participants enrolled by PRSS through community engagement/outreach (not specifically through treatment programs or sober living).

e Participants enrolled in a previous wearable study which were transferred over to the current study.

**Table 2:** Substance use characteristics.

	All participants enrolled (*n* = 229)			Randomized to PRSS intervention vs. SoC (≥30% compliant)									
				Overall sample (*n* = 63)			PRSS intervention (*n* = 26)			SoC (*n* = 37)			*p*
	Ever use (*n*, %)	Recent Use^a^ (*n*, %)	Age at first use^b^	Ever use (*n*, %)	Recent Use^a^ (*n*, %)	Age at first use^b^	Ever Use (*n*, %)	Recent Use^a^ (*n*, %)	Age at first use^b^	Ever Use (*n*, %)	Recent Use^a^ (*n* %)	Age at first use^b^	
Opioids													
* Yes*	209 (91.7)	6 (2.6)	23.8 (6.4)	58 (92.1	2 (3.2	20.5	25 (96.2)	1 (3.8)	25 (N/A)	33 (89.2)	1 (2.7)	16 (N/A)	–
* No*	19 (8.3)	140 (61.4)		5 (7.9)	39 (61.9)		1 (3.8)	14 (53.8)		4 (10.8)	25 (67.6)		
*Missing*	0	82 (36.0)		0	22 (34.9)		0	11 (42.3)		0	11 (29.7)		
Meth													
* Yes*	192 (84.2)	8 (3.5)	22.9 (9.9)	55 (87.3)	3 (4.8)	20.3 (8.4)	23 (88.5)	1 (3.8)	15 (N/A)	32 (86.5)	2 (5.4)	23 (9.9)	–
* No*	36 (15.8)	179 (78.5)		8 (12.7)	52 (82.5)		3 (11.5)	22 (84.6)		5 (13.5)	30 (81.1)		
*Missing*	0	41 (18.0)		0	8 (12.7)		0	3 (11.5)		0	5 (13.5)		
Cocaine													
* Yes*	214 (93.9)	5 (2.2)	28.2 (8.0)	60 (95.2)	1 (1.6)	N/A	25 (96.2)	1 (3.8)	N/A	35 (94.6)	0	N/A	–
* No*	14 (6.1)	207 (90.8)		3 (4.8)	59 (93.7)		1 (3.8)	24 (92.3)		2 (5.4)	35 (94.6)		
*Missing*	0	16 (7.0)		0	3 (4.8)		0	1 (3.8)		0	2 (5.4)		
Benzos													
* Yes*	152 (66.7)	1 (0.4)	20 (N/A)	48 (76.2)	0	N/A	19 (73.1)	0	N/A	29 (78.4)	0	N/A	–
* No*	75 (32.9)	151 (66.2)		15 (23.8)	48 (76.2)		7 (26.9)	19 (73.1)		8 (21.6)	29 (78.4)		
*Missing*	1 (0.4)	6 (33.3)		0	15 (23.8)		0	7 (26.9)		0	8 (21.6)		
Cannabis													
* Yes*	225 (98.7)	21 (9.2)	15 (4.8)	63 (100.0)	8 (12.7)	15.1 (4.2)	26 (100.0)	5 (19.2)	16 (4.8)	37 (100.0)	3 (8.1)	13.7 (3.2)	0.45
* No*	3 (1.3)	203 (89.0)		0	55 (87.3)		0	21 (80.8)		0	34 (91.9)		
*Missing*	0	4 (1.8)		0	0		0	0		0	0		
Alcohol													
* Yes*	215 (94.3)	21 (9.2)	12.9 (2.6)	60 (95.2)	8 (12.7)	12 (2.2)	24 (92.3)	2 (7.7)	13.5 (2.1)	36 (97.3)	6 (16.2)	11.5 (2.2)	0.38
* No*	5 (2.2)	197 (86.4)		2 (3.2)	54 (85.7)		1 (3.8)	23 (88.5)		1 (2.7)	31 (83.8)		
*Missing*	8 (3.5)	10 (4.4)		1 (1.6)	1 (1.6)		1 (3.8)	1 (3.8)		0	0		

Benzos, benzodiazepines; Meth, methamphetamine; PRSS, peer recovery support specialist; Pt, participant; SoC, standard of care; Tx, treatment.

a Recent use defined as any use reported in the 30 days prior to enrollment.

b Age at first use represents Mean (SD) for those with recent use.

**Table 3:** Distribution of the number of days both wearable device (Oura Ring) and EMA (RNI Health app) were worn/completed (*n* = 229).

	All participants enrolled (*n* = 229)	PRSS intervention (*n* = 108)	SoC (*n* = 121)	*p*
Baseline (0–90 days)				
Total # of Days Compliant * Mean (SD)*	40.8 (28.1)	40.1 (28.6)	41.4 (27.7)	0.81
Compliant days—range^a^				
1–26 days	82 (35.8%)	40 (37.0%)	42 (34.7%)	0.92
27–53 days	53 (23.1%)	24 (22.2%)	29 (24%)	
≥54 days	94 (41.0%)	44 (40.8%)	50 (41.3%)	
Phase 1 (91–180 days)				
Total # of Days Compliant * Mean (SD)*	29.8 (29.7)	28.8 (29.8)	30.7 (29.8)	0.60
Compliant days—range^a^				
1–26 days	118 (51.5%)	57 (52.8%)	61 (50.4%)	0.69
27–53 days	48 (21.0%)	20 (18.5%)	28 (23.1%)	
≥54 days	63 (27.5%)	31 (28.7%)	32 (26.5%)	
Phase 2 (181–270 days)				
Total # of Days Compliant * Mean (SD)*	21.3 (26.8)	18.6 (26.3)	23.7 (27.1)	0.12
Compliant days—range^a^				
1–26 days	146 (63.8%)	75 (69.4%)	71 (58.7%)	0.22
27–53 days	50 (21.8%)	19 (17.6%)	31 (25.6%)	
≥54 days	33 (14.4%)	14 (13.0%)	19 (15.7%)	

PRSS, peer recovery support specialist; SoC, standard of care.

a Data reflect the number of individuals compliant for each of the three range bands and the reflective percentage.

Of the 229 participants, 108 provided at least one datapoint (either Oura or EMA) during each phase (Baseline, Phase 1, and Phase 2). Seventy-eight participants provided ≥30% of EMA and Oura data across Baseline, Phase 1, and Phase 2. Of these 78 participants, 15 were excluded because they erroneously received calls during the baseline period, leaving 63 for inclusion in the analyses. Twenty-six were randomized to the PRSS Intervention arm, and 37 were randomized to the SoC arm (Figure 1b). Demographic and substance use characteristics were statistically similar for those who were ≥30% compliant vs. those who were not (all *p*'s > 0.1).

### 3.2 Models developed for predicting risk factors of drug use recurrence

The four predictive models included anxiety, depression, stress, and craving. Both generalized (for the entire sample) and personalized (for each unique participant based on individual responses) models detected data anomalies. In total, there were 225,374 generalized predictions and 84,240 personalized predictions, representing 72.8% and 27.2% of the total predictions, respectively. The generalized model correctness estimates were the following: anxiety (90.74%), depression (92.7%), stress (93.8%), and craving (92.5%). The individual model correctness was as follows: anxiety (90.4%), depression (92.2%), stress (91.4%), and craving (97.3%). Daily EMA responses were generally better predictors than Oura data or monthly EMA responses.

### 3.3 PRSS intervention

Demographic information for those who were ≥30% compliant (*n* = 63) and randomized to the PRSS Intervention (*n* = 26) and SoC (*n* = 37) arms are included in Table 1 and substance use characteristics are included in Table 2 (for all participants randomized and each randomization arm separately). Overall, participants who were randomized were similar to the entire sample of 229 participants enrolled and were equally distributed based on sex (male: 52%, female: 48%), were predominantly White (91%), were 40.6 ± 8.2 (mean ± SD) years of age, and >90% reported a history of opioid, stimulant, cannabis, and alcohol use. There were no differences in demographic or substance use characteristics between the PRSS Intervention and SoC arms (all *p*'s > 0.05).

As described in the methods, participants in the PRSS Intervention arm received calls when predictive models indicated increased risk of DUR. The PRSS made ∼21 calls per week on average over the course of the enrollment period (median = 20; range = 0–65). The average number of calls made to each unique participant across the duration of enrollment was ∼20 (median = 15; range = 1–53). Over the course of the study, the PRSS made a total of 483 call attempts for a unique flag, and of these, participants answered the first call of the PRSS on 175 occasions (36% of the time). A voicemail was left on 206 occasions, and there were 109 instances when a voicemail could not be left (e.g., phone number was incorrect, disconnected, or voicemail was full). A breakdown of the number of calls made by the PRSS and the calls answered by the participant can be found in Table 4.

**Table 4:** Number of calls sent to and answered by participants with optimal compliance in the PRSS intervention arm.

	PRSS intervention arm *Cohort with optimal compliance* (*n* = 26)	
	Calls sent^a^	Calls answered^b^
Overall (Phase 1 and 2 combined)		
# of calls per participant^c^	13.4 (9.4)	5.1 (5.3)
Calls per pt—range		
1–5 calls	4 (15.4%)	16 (61.5%)
6–10 calls	4 (15.4%)	6 (23.1%)
11–20 calls	15 (57.7%)	3 (11.5%)
21 + calls	3 (11.5%)	1 (3.9%)
Phase 1		
# of call per pt^c^	6.2 (3.9)	2.6 (2.4)
Calls per pt—range		
1–5 calls	13 (50.0%)	23 (88.5%)
6–10 calls	8 (30.8%)	3 (11.5%)
11–20 calls	5 (19.2%)	0
21 + calls	0	0
Phase 2		
# of call per pt^c^	7.2 (7.4)	2.5 (3.5)
Calls per pt—Range		
1–5 calls	14 (53.8%)	22 (84.6%)
6–10 calls	6 (23.1%)	3 (11.5%)
11–20 calls	5 (19.2%)	1 (3.9%)
21 + calls	1 (3.9%)	0

PRSS, peer recovery support specialist; Pt, participant.

a Numbers reflect the number of calls sent and the representative percentage of the total calls made.

b Numbers reflect the total numbers of calls answered and the representative percentage of total calls answered. Total number of calls answered do not align with the total number of calls sent (e.g., participants who had 6–10 calls sent, may have only answered 1–5 of those calls).

c Values reflect Mean (SD).

**Figure 2:**
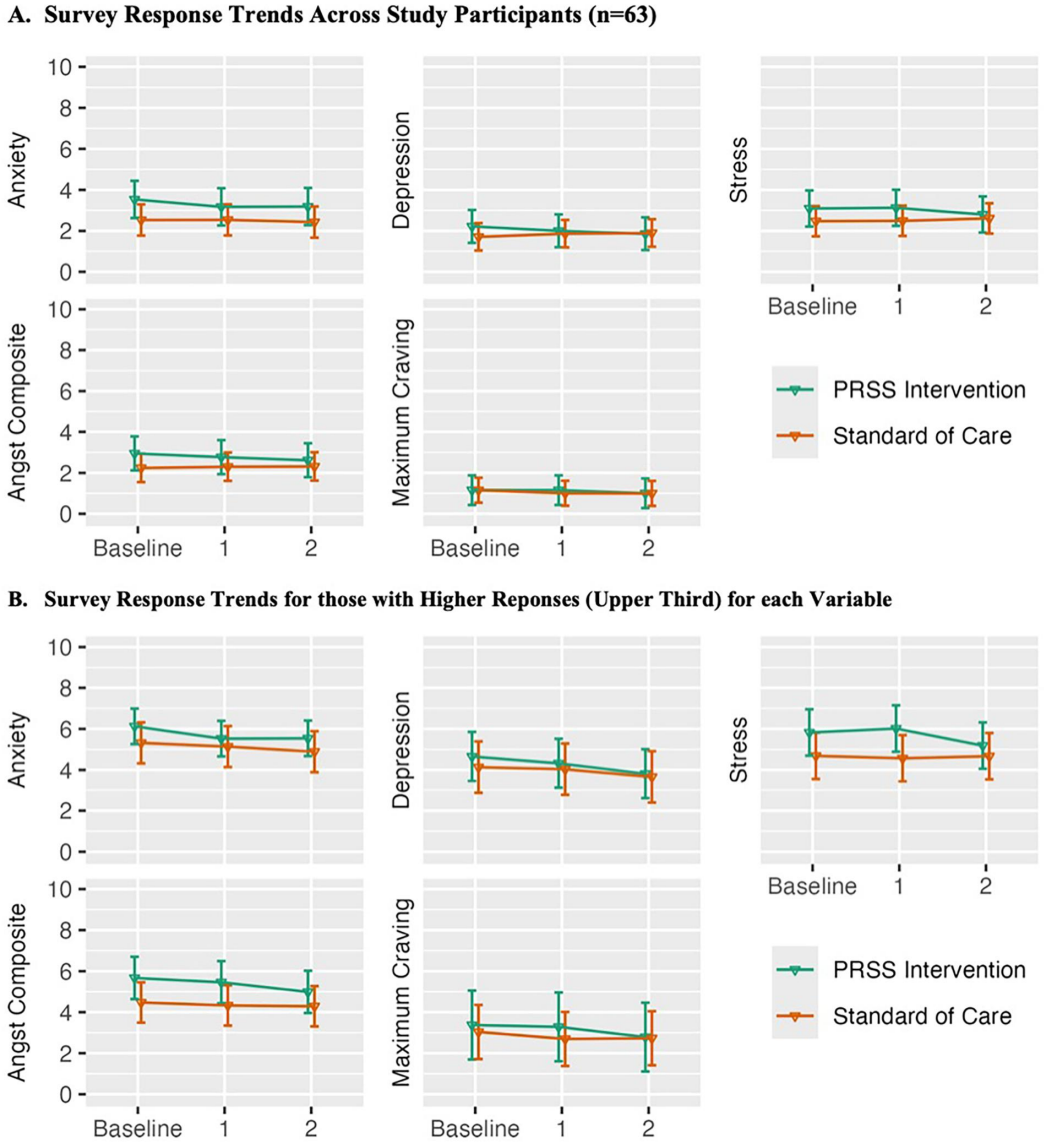
**(A)** Survey response trends across study participants (*n* = 63); **(B)** survey response trends for those with higher reponses (upper third) for each variable.

The average response for each survey (anxiety, depression, stress, angst composite, and maximum craving) across the different phases is presented in Figure 2A. With 13,485 observations across 63 subjects available for anxiety, depression, stress, angst composite, and maximum craving, all main effects across study time points, as well as all interaction effects between the SoC and PRSS Intervention arms, were found to be statistically significant. For the main effect of timepoint, there was a modest but significant decrease in the average scores as the study progressed (suggesting the positive impact of study participation). The interaction effects were significant due to the differing patterns between the SoC and PRSS Intervention arms over time. For each of the five variables, the PRSS Intervention arm had a lower average score in Phase 2 compared to their Baseline. Conversely, for the SoC arm, depression, stress, and angst composite levels slightly increased during Phase 2 relative to their Baseline, suggesting that the PRSS calls appeared to benefit the participants with respect to these variables. Out of the five variables, the change in maximum craving from Baseline to Phase 2 was most similar between the PRSS Intervention and SoC arms, but the interaction effect remained significant (as the decrease occurred at different timepoints for each arm, during Phase 1 for SoC and during Phase 2 for PRSS Intervention arm).

To further elucidate any potential changes as a function of the PRSS Intervention and to address floor effects (those who did not report higher responses on any of those variables), the highest third of responders for each of those variables was extracted and included in Figure 2B. The minimum response values and range for each of the five variables, along with the significance, is shown in Table 5. The breakdown of responses for the highest third, middle third, and lower third responses can be found in Supplementary Figure S1. For those participants with higher average response values, all main effects for timepoint are highly significant, with a more consistent decrease from Baseline to Phase 2. The interaction effects are also all significant, though in some cases (e.g., anxiety, depression) not as strongly as with the overall analysis. The most notable change across variables involves the stress variable. At Baseline and Phase 1, the PRSS Intervention arm has a higher average relative to the SoC arm; however, there is a decrease in the PRSS arm during Phase 2, and the difference between arms becomes minimal. Similar trends are also observed with depression and the angst composite, though not to the same degree as with the stress variable. This may not be as clear, however, as the gap by Phase 2 is still larger than with stress.

**Table 5:** Survey response characteristics and differences for those with higher responses for each variable.

Category	Upper third minimum Score	# of Participants in the upper third (n)		*p*-values		
		PRSS intervention	SoC	Arm main	Timepoint main	Interaction
Anxiety	4.04	12	9	0.339	<0.001	0.010
Depression	2.53	11	10	0.701	<0.001	0.027
Stress	3.38	10	10	0.189	<0.001	<0.001
Angst	3.07	10	11	0.153	<0.001	<0.001
Craving	0.93	8	13	0.750	<0.001	0.001

PRSS, peer recovery support specialist; SoC, standard of care.

## 4 Discussion

Sixty-three of 229 participants in various stages of SUD recovery participated for at least 30% of the first 270 days of a 450-day research study involving the use of a wearable device to monitor biometric signals and the use of a phone-based application (RNI Health app) to complete questionnaires regarding mood, pain, and cravings. When models predicted anomalies, a PRSS staff member was notified to reach participants to offer support and to connect them to services. Statistically significant reductions were noted in anxiety, depression, stress, angst, and cravings in the PRSS intervention arm relative to the SoC arm, though the extent of the differences was modest, therefore likely tempering the clinical significance. Important aspects related to feasibility included difficulties with learning how to use the RNI Health app, the high burden of daily entries with the RNI Health app, limited battery life of the wearable devices, loss of wearable devices, and the high burden of responding by phone to numerous alerts generated by the algorithms.

Despite the informative findings, there are some important limitations of this study that must be noted. The study was originally designed to include weekly urine toxicology for confirmation of substance use/non-use and to verify the accuracy of participant self-report of substance use/non-use. However, as a result of COVID-19 restrictions and the subsequent transition to telehealth, participants were unable to attend the clinic in person on a regular basis. Consequently, weekly urine toxicology assessments were not conducted. Instead, participants were randomly requested to come to the clinic so that urine toxicology could be performed as part of their clinical care. In this study, we did not examine DUR as an outcome; however, future studies will need to address reliable means for detecting DUR. Another study limitation is the relatively small sample size, partially due to high rates of attrition and low compliance. High rates of attrition are common in studies involving individuals from high-risk populations. Reasons for poor compliance in the current study included a short battery life for the Oura rings, loss of rings, and a high burden associated with daily app entries. When compliance is low, model performance is negatively impacted because there is less data for both the training and testing sets leading to higher prediction variance due to overfitting (“memorizing” the training data rather than learning the underlying patterns, and thus not generalizing well to new data in the test set). Finally, we did not have access to medical records for many of the study participants, limiting the number and type of variables that could have been included in predictive models. Medical records also could have served as an additional sources of urine drug screen results. Another limitation we faced was the inability to distinguish between anomaly-based alerts and random alerts. Our goal was to evaluate differences in outcomes for participants receiving each type of alert, alongside check-ins by the blinded PRSS. However, due to a technical issue with the database, we lost the identifiers that indicated whether alerts were random or anomaly-generated. Although we know that 75% of the alerts were anomaly-generated, we could not differentiate these from the random alerts, preventing the completion of this analysis. We have since corrected the database export process to enable this separation in future studies.

Future studies should address several challenges in order to inform successful prevention interventions for SUD. Ideally, wearable devices should be inexpensive and easily replaced if they are lost or malfunction. They should have a long battery life and be easy to synchronize. The Oura devices in this study were Generation 3; devices now available are Generation 4 and have a longer battery life and improved sensor capabilities (which should also reduce motion artifact which was not explicitly addressed in the preprocessing performed in the current study). If the window of time between a signal of possible relapse and the actual relapse itself is narrow, it would be important to trigger and implement interventions quickly. Achieving this is challenging for a number of reasons. In our study, the burden on the PRSS staff was high. If PRSS are to be part of the intervention, a program would require a sufficient number of trained PRSS staff to be available for extended hours; the cost of such a program could be prohibitive. In addition, the burden on participants from completing daily questionnaires was high and compliance was low. Models relying on other factors that do not include frequent questionnaire entries (such as data from wearables and medical history) may be needed to optimize the efficacy of the intervention. Developing accurate models to predict relapse also requires accurate information about the timing and type of relapse. This can be challenging when patients either do not disclose their substance use to research staff, when urine drug screens are obtained infrequently, or when urine screen results are erroneous (either false positives or false negatives).

In the future, studies should not only include a larger sample but also acquire EMA-APP and wearable device data over a longer time-period. It would be ideal to capture multiple DURs followed by sustained periods of abstinence to determine the consistency of the potential predictors identified in this study. It may be necessary to provide incentives and more frequent engagement with participants, including convenient opportunities for frequent drug testing. In conclusion, despite the limitations noted above, this study highlights the potential of developing models to predict risk factors for DUR via wearable devices and daily subjective reporting (EMA). This study also demonstrates the potential utility of implementing a PRSS intervention once the model detects that the individual may be at a higher risk for experiencing a DUR. There are several challenges noted that impact model development and subsequent PRSS intervention, the most prominent involving patient compliance and attrition, which must be improved in order to optimize model accuracy and implementation.

## Data Availability

The raw data supporting the conclusions of this article will be made available by the authors, without undue reservation.

## References

[1] Key substance use and mental health indicators in the United States: Results from the 2024 National Survey on Drug Use and Health (HHS Publication No. PEP23-07-01-006, NSDUH Series H-58). Center for Behavioral Health Statistics and Quality, Substance Abuse and Mental Health Services Administration.

[2] Ahmad FB Cisewski JA Rossen LM Sutton P. Provisional drug overdose death counts. Natl Center Health Stat. (2026). 10.15620/cdc/20250305008

[3] Sordo L Barrio G Bravo MJ Indave BI Degenhardt L Wiessing L. Mortality risk during and after opioid substitution treatment: systematic review and meta-analysis of cohort studies. Br Med J. (2017) 357:j1550. 10.1136/bmj.j1550 28446428 PMC5421454

[4] Weiss RD Potter JS Fiellin DA Byrne M Connery HS Dickinson W. Adjunctive counseling during brief and extended buprenorphine-naloxone treatment for prescription opioid dependence: a 2-phase randomized controlled trial. Arch Gen Psychiatry. (2011) 68(12):1238–46. 10.1001/archgenpsychiatry.2011.121 22065255 PMC3470422

[5] Nunes EV Gordon M Friedmann PD Fishman MJ Lee JD Chen DT. Relapse to opioid use disorder after inpatient treatment: protective effect of injection naltrexone. J Subst Abuse Treat. (2018) 85:49–55. 10.1016/j.jsat.2017.04.016 28473233 PMC5755382

[6] Williams AR Samples H Crystal S Olfson M. Acute care, prescription opioid use, and overdose following discontinuation of long-term buprenorphine treatment for opioid use disorder. Am J Psychiatry. (2020) 177(2):117–24. 10.1176/appi.ajp.2019.19060612 31786933 PMC7002204

[7] Lyons RM Yule AM Schiff D Bagley SM Wilens TE. Risk factors for drug overdose in young people: a systematic review of the literature. J Child Adolesc Psychopharmacol. (2019) 29(7):487–97. 10.1089/cap.2019.0013 31246496 PMC6727478

[8] Khezri M Rahman F Alexander M Zielinski MJ Bunting AM. Increased risk of non-fatal overdose associated with broad adverse childhood experiences among people who use drugs in New York City: a latent class analysis. Am J Drug Alcohol Abuse. (2025) 51(5):667–76. 10.1080/00952990.2025.2563052 41160809 PMC13001614

[9] Segel J Chu W Ferguson A. Preventing Overdose Deaths During High Risk Transition Periods. University Park, PA: Research-to-Policy Collaboration Briefs, Penn State Social Science Research Institute (2024). Available online at: https://evidence2impact.psu.edu/resources/preventing-overdose-deaths-during-high-risk-transition-periods/ (Accessed November 1, 2025).

[10] SAMHSA Incorporating Peer Support Into Substance Use DisorderTreatment Services 2023. Substance Abuse and Mental Health Services Administration. Incorporating Peer Support Into Substance Use Disorder Treatment Services. Treatment Improvement Protocol (TIP) Series 64. Publication No. PEP23-02-01-001. Rockville, MD: Substance Abuse and Mental Health Services Administration (2023).

[11] Day E Pechey LC Roscoe S Kelly JF. Recovery support services as part of the continuum of care for alcohol or drug use disorders. Addiction. (2025) 120(8):1497–520. 10.1111/add.16751 39873444 PMC12215296

[12] Hoeppner BB Simpson HV Weerts C Riggs MJ Williamson AC Finley-Abboud D. A nationwide survey study of recovery community centers supporting people in recovery from substance use disorder. J Addict Med. (2024) 18(3):274–81. 10.1097/ADM.0000000000001285 38426533 PMC11150096

[13] D’Souza JM Wardle M Green CE Lane SD Schmitz JM Vujanovic AA. Resting heart rate variability: exploring associations with symptom severity in adults with substance use disorders and posttraumatic stress. J Dual Diagn. (2019) 15(1):2–7. 10.1080/15504263.2018.1526431 30418104 PMC6511322

[14] Fletcher RR Tam S Omojola O Redemske R Kwan J. Wearable sensor platform and mobile application for use in cognitive behavioral therapy for drug addiction and PTSD. Annu Int Conf IEEE Eng Med Biol Soc. (2011) 2011:1802–5. 10.1109/IEMBS.2011.6090513 22254678

[15] Mahoney JJ Finomore VS Marton JL Ramadan J Hodder SL Thompson-Lake DGY. Identifying biomarkers of drug use recurrence using wearable device technologies and phone applications. Drug Alcohol Depend. (2023) 249:110817. 10.1016/j.drugalcdep.2023.110817 37331302 PMC10416187

[16] Carreiro S Chintha KK Shrestha S Chapman B Smelson D Indic P. Wearable sensor-based detection of stress and craving in patients during treatment for substance use disorder: a mixed methods pilot study. Drug Alcohol Depend. (2020) 209:107929. 10.1016/j.drugalcdep.2020.107929 32193048 PMC7197459

[17] Carreiro S Ramanand P Akram W Stapp J Chapman B Smelson D. Developing a wearable sensor-based digital biomarker of opioid dependence. Anesth Analg. (2025) 141(2):393–402. 10.1213/ANE.0000000000007244 39413034 PMC12000379

[18] Carreiro S Ramanand P Taylor M Leach R Stapp J Sherestha S. Evaluation of a digital tool for detecting stress and craving in SUD recovery: an observational trial of accuracy and engagement. Drug Alcohol Depend. (2024) 261:111353. 10.1016/j.drugalcdep.2024.111353 38917718 PMC11260438

[19] Preston KL Kowalczyk WJ Phillips KA Jobes ML Vahabzadeh M Lin J-L. Before and after: craving, mood, and background stress in the hours surrounding drug use and stressful events in patients with opioid-use disorder. Psychopharmacology. (2018) 235(9):2713–23. 10.1007/s00213-018-4966-9 29980821 PMC6119104

