# Supplementary material for: Identifying risk factors for drug use recurrence with ecological momentary assessment, wearable technologies, and machine learning: a feasibility trial of peer recovery support specialist intervention
Source: Front Digit Health. 2026 Jul 3;8:1744937. doi: 10.3389/fdgth.2026.1744937 (PMC13375731; doi:10.3389/fdgth.2026.1744937)

**Identifying Risk Factors for Drug Use Recurrence with Ecological Momentary Assessment, Wearable Technologies, and Machine Learning: A Feasibility Trial of Peer Recovery Support Specialist Intervention**

James J. Mahoney III^1-3*^, Victor S. Finomore^3^, Jennifer L. Marton^3^, Lucinda J. England^2,3^, Sara McFoy^3^, Danielle Romanoff^3^, Jad Ramadan^3^, Anahita Zarei^3^, Amer Mahyoub^3^, Jessie Jones^2,3^, James H. Berry^2,3^, Steven D. Shirk^3^, Manish Ranjan^3,4^, Ali R. Rezai^3,4^

**Table of contents**

**Supplemental Methods.** Development of Personalized and Generalized Models for Anomaly Detection

**Supplemental Figure S1.** Survey Response Trends Across for Highest, Middle, and Lowest Responders for Each Variable

**Supplemental Methods: Development of personalized and generalized models for anomaly detection**

Four personalized models for predicting craving, anxiety, stress, and depression were developed using deep learning architectures. This approach ensured that patients received timely personalized alerts when their predicted risk scores indicate a significant deviation from their established baseline (defined as >1.5 standard deviations from their previous 28-day average). The following summarizes the six stages for model development and implementation:

1. *Data Acquisition and Merging:* Data related to daily surveys (e.g., stress, craving, depression daily scores), Oura ring metrics, and various monthly survey scores were compiled and integrated within a Redshift database.
2. *Feature Engineering for Time-Series Modeling:* New features were generated by shifting relevant input features by one day to capture temporal dependencies and creating a 2-day rolling window. The data were then split into training sets (described in #3) and testing sets (referred to as the “hold-out test set” and described in #4 below).
3. *Personalized Model Training:* A custom function was implemented to train a separate prediction model for each individual participant. As part of this function, the preprocessing pipeline handles missing data imputation, standardization, and reshaping the input into the correct format for Long Short-Term Memory (LSTM) and Gated Recurrent Unit (GRU) models, that are effective in relatively short-sequence prediction tasks. LSTM/GRU models are types of recurrent neural networks (RNNs) that help machines understand and process sequences of data. The models have been hyper-tuned and validated by using time series split cross-validation, to ensure proper evaluation of hyperparameters while respecting the temporal order of the data. We used negative median absolute error as the scoring metric.
4. *Model’s performance assessment:* After training the model using an individual’s specific training data, the model's performance was assessed using a hold-out test set. In machine learning research, a hold‐out test set is defined as the subset of data that is deliberately withheld from the model during both the training and hyperparameter‐tuning phases. In order to get accurate results that reflect real-world performance, it is critical to ensure that the hold-out test set is sufficiently large enough and representative of the domain being assessed. After a model has been fully specified and fitted on its training data, its predictive performance is evaluated on this reserved hold-test set. Because the model has never “seen” these examples before, metrics computed on the hold-out test set (such as accuracy, precision, recall or mean squared error) provide an unbiased estimate of how well the model is likely to generalize to novel data.
5. *Comparison and Selection:* Both GRU and LSTM models were trained for each individual. A comparison was made between the GRU and LSTM models’ performance metrics for each participant and a model of choice is determined based on the best metrics.

**Supplemental Figure S1**: Survey Response Trends Across for Highest, Middle, Lowest Responders for Each Variable


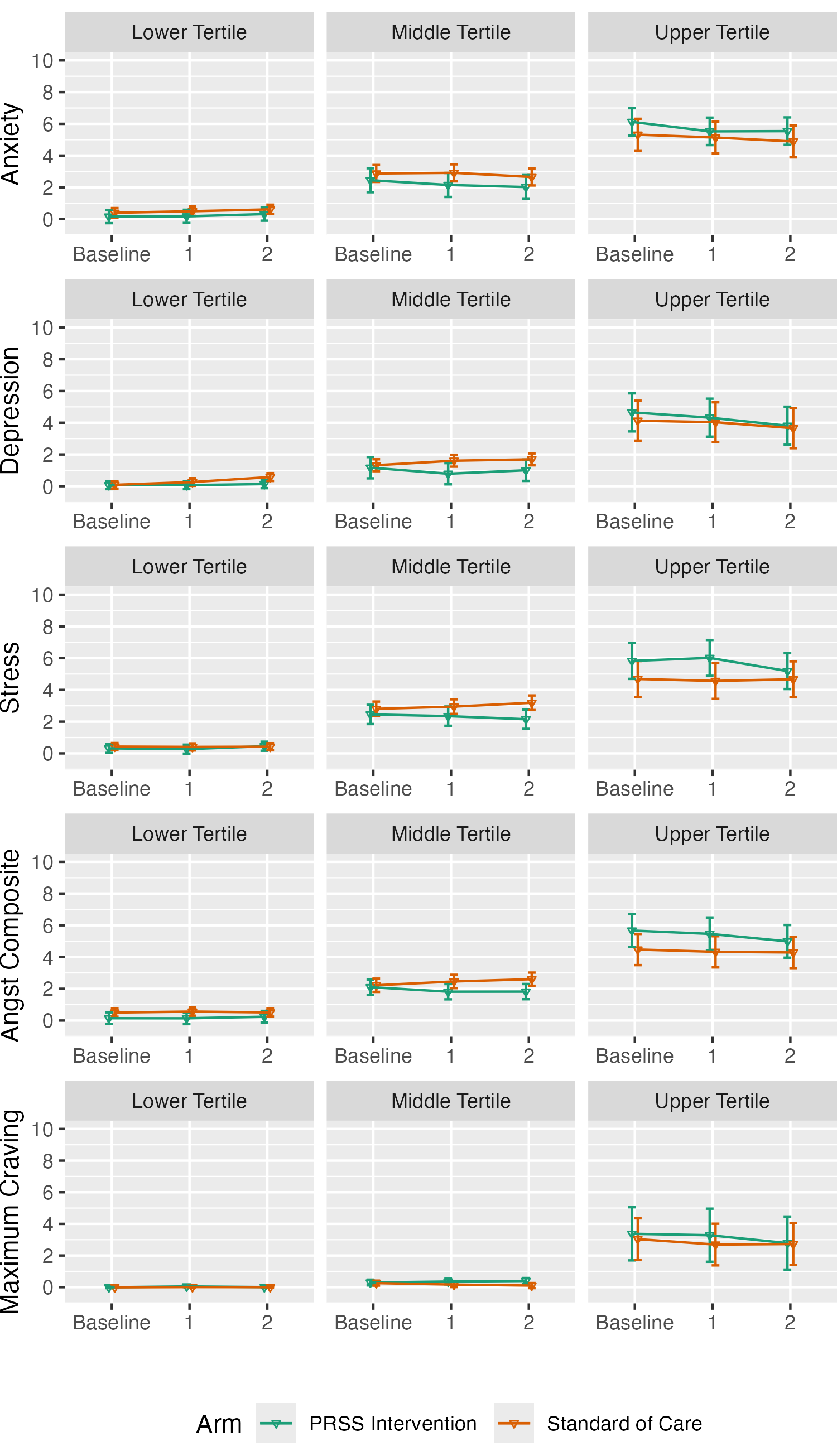

Supplement: Supplementary file 1 [file Datasheet1.docx]
